# Curcumin and berberine co-loaded liposomes for anti-hepatocellular carcinoma therapy by blocking the cross-talk between hepatic stellate cells and tumor cells

**DOI:** 10.3389/fphar.2022.961788

**Published:** 2022-09-14

**Authors:** Jingliang Wu, Cuiping Qi, Hao Wang, Qing Wang, Jingui Sun, Jinping Dong, Guohua Yu, Zhiqin Gao, Bo Zhang, Guixiang Tian

**Affiliations:** ^1^ School of Nursing, Weifang University of Science and Technology, Weifang, China; ^2^ School of Nursing, Weifang Medical University, Weifang, China; ^3^ Department of Oncology, Weifang People’s Hospital, Weifang, China; ^4^ School of Bioscience and Technology, Weifang Medical University, Weifang, China; ^5^ School of Pharmacy, Weifang Medical University, Weifang, China

**Keywords:** curcumin, berberine, combination therapy, delivery, liposomes

## Abstract

Cancer-associated fibroblasts (CAFs) are a major component of the tumor microenvironment (TME). In hepatocellular carcinoma (HCC), quiescent hepatic stellate cells (HSCs) could be activated to become CAFs, which play a critical role in tumor progression and drug resistance. Therefore, recent efforts have been focused on combining anti-HSC and pro-apoptotic activities to improve anti-tumor efficacy of drugs. In this study, glycyrrhetinic acid and hyaluronic acid–modified liposomes (GA-HA-Lip) were prepared for co-delivery of curcumin (CUR) and berberine (BBR) for the treatment of HCC. Furthermore, we established the LX-2+BEL-7402 co-cultured cell model and implanted the m-HSCs+H22 cells into a mouse to evaluate the anti-tumor effect of CUR&BBR/GA-HA-Lip both *in vitro* and *in vivo*. The results showed that CUR&BBR/GA-HA-Lip could accumulate in tumor tissues and be taken up by HSCs and BEL-7402 cells simultaneously. Compared with free CUR, the combination therapy based on GA-HA-Lip exhibits stronger pro-apoptotic and anti-proliferation effect both *in vitro* and *in vivo*. The anti-tumor mechanistic study revealed that CUR&BBR/GA-HA-Lip could inhibit the activation of HSCs and restrain drug resistance of tumor cells. In summary, CUR&BBR/GA-HA-Lip could be a promising nano-sized formulation for anti-tumor therapy.

## Introduction

Hepatocellular carcinoma (HCC) is one of the deadliest human malignancies worldwide (Bray et al., 2018; Dietrich et al., 2020). The current therapy cannot effectively inhibit tumor development and drug resistance (Zhou et al., 2018; Anwanwan et al., 2020; Thakkar et al., 2020). According to the annual report of the World Health Organization, more than one million patients will die from HCC in 2030 (Shek et al., 2021). Recent research works have revealed that hepatic stellate cells (HSCs) are located in the tumor microenvironment (TME) (Azzariti et al., 2016; Brodt, 2016; Hu et al., 2019). In the TME, tumor cells secrete various cytokines, such as TGF-β (Caballero-Díaz et al., 2020) and PDGF (Kocabayoglu et al., 2015; Anderson and Simon, 2020), to promote the activation and proliferation of HSCs, and the activated HSCs (aHSCs) induce drug resistance and epithelial–mesenchymal transition (EMT) by producing pro-tumor factors such as HGF, IGF, and others (Camblin et al., 2018; Chen et al., 2021a; Östman, 2017; Rhee et al., 2018). Numerous studies demonstrated that the cross-talk between aHSCs and tumor cells play an important role in tumor development (Ruan et al., 2020; Myojin et al., 2021). Therefore, the combination therapy with pro-apoptotic and anti-aHSC activities might be a potential strategy to inhibit drug resistance of tumor cells.

Recent research work indicated that TGF-β could induce a shift from quiescent HSCs to CAF phenotype (Chen et al., 2021b; Wang et al., 2019; Zhang et al., 2017). The aHSCs could upregulate the expression of marker proteins of cancer-βassociated broblasts (CAFs), such as α-smooth muscle actin (α-SMA) (Hu et al., 2017; Miao et al., 2020) and fibroblast-activated protein (FAP) (Kaps and Schuppan, 2020; Loh et al., 2021), and produce extracellular proteins, such as collagen I, collagen III, and fibronectin, to form extracellular matrix (ECM) deposition, which could decrease the sensitivity of chemotherapeutic drugs (Multhaupt et al., 2016). Blocking HSC activation is an effective way to inhibit the development of HSCs. Previous studies have shown that berberine (BBR), an isoquinoline alkaloid extracted from the Chinese herb, *Coptis chinensis*, and other *Berberis* plants, has exhibited a variety of pharmacological properties including anti-inflammatory, anti-diabetic, and anti-cancer activities (Allijn et al., 2017; Song et al., 2020). Moreover, it is worth noting that BBR could inhibit TGF-β–induced myofibroblast differentiation in the fibrotic conditions by activating the AMPK signaling pathway (Fan et al., 2020). Based on this, we hypothesized that the combination therapy of BBR and pro-apoptotic drug could improve anti-tumor efficacy by blocking the tumor–aHSCs cross-talk and inducing apoptosis of tumor cells.

Liposomes have the characteristics of nano-scale properties, similar biofilm structure, good biocompatibility, low systemic toxicity, long half-life, and so on, which has been proved to be a promising anti-tumor drug (Xie et al., 2020; Gao et al., 2022). Since the first liposome-encapsulating drug entered the clinical trial stage in 1985 (Morgan et al., 1985), more than 40 liposome-loaded drugs have been successfully marketed or are in different clinical research stages (Allen et al., 2013). In our previous research, we found that glycyrrhetinic acid (GA) receptor was overexpressed on HCC cells, and GA-modified liposomes (GA-Lip) could obviously improve drug internalization *via* GA-R–mediated endocytosis (Jiang et al., 2019; Qi et al., 2021). However, GA-Lip could not effectively target aHSCs due to their low expression level of the GA receptor. Recent research revealed that CD44, a cell surface glycoprotein, was overexpressed in aHSCs (Qian et al., 2018). It was well known that hyaluronic acid (HA) was a potent ligand for active-targeting delivery of drugs to the CD44^+^ cells (Deng et al., 2017; Lv et al., 2018). Based on that, the liposomes modified by HA and GA molecules might target aHSCs and tumor cells by HA/CD44- and GA/GA receptor-mediated endocytosis, respectively.

In this study, novel GA&HA-modified liposomes were prepared for co-delivery of BBR and CUR. The co-loaded liposomes possess the following advantages: 1) inhibit activation of HSCs, 2) reduce drug resistance of HCC by blocking the cross-talk between aHSCs and tumor cells, and 3) improve anti-tumor effect by HA/CD44- and GA/GA receptor-mediated drug internalization.

To verify the effectiveness of the combination therapy, anti-tumor efficacy was evaluated both *in vitro* and *in vivo*. The *in vitro* cytotoxicity and cellular uptake of different drug formulations were performed in two cell models: tumor cells and “tumor+HSCs” mixed cells. Moreover, “H22+m-HSC” co-bearing mice were established to evaluate the anti-tumor effect of the co-loaded liposomes.

## Materials and methods

### Materials

CUR (MW 368.38 Da), BBR (MW 336.36 Da), and cholesterol (Chol) were purchased from Aladdin Chemistry Co., Ltd. (China). L-α-phosphatidylcholine (PC), DSPE-PEG_2000_-NH_2_, and DSPE-PEG_2000_-FITC were obtained from Xi’an Ruixi Bio-Tech Co., Ltd. RPMI 1640 medium, Dulbecco’s Modified Eagle’s Medium (DMEM), and fetal bovine serum (FBS) were purchased from Beijing Solarbio Bio-Tech Co., Ltd. (China). GA (MW 470.69 Da) was obtained from Shanxi Fujie Pharmaceutical Co., Ltd. (China). 1,1′-Dioctadecyl-3,3,3,3-tetramethyl indotricarbocyanine iodide (DiR) was acquired from Beijing Fanbo Biochemicals (China). 3-(4,5-Dimethylthiazol-2-yl)-2,5-diphenyl tetrazolium bromide (MTT) and 4′,6-diamidine-2′-phenylindole dihydrochloride (DAPI) were purchased from Sigma-Aldrich. A CFSE-Cell Labeling Kit was obtained from Abcam (Cambridge, MA, United States). All other reagents used in this study were of analytical grade.

### Cell lines and animals

Human HCC cells (BEL-7402), human-derived HSCs (LX-2), mouse HCC cells (H22), and mouse HSCs (m-HSCs) were obtained from Beijing BeNa Culture Collection (BNCC, China). BEL-7402 and LX-2 cells were cultured in RPMI 1640 or DMEM with 10% FBS and 1% penicillin/streptomycin, respectively, in a cell incubator (37°C 5% CO_2_).

Female BALB/c mice (6–7 weeks) were purchased from Ji’nan Pengyue Experimental Animal Breeding Co., Ltd. (China). The *in vivo* anti-tumor studies were approved by the Animal Ethics Committee of Weifang Medical University (2019-046), and the care and handling procedures strictly complied with “Guide for the Care and Use of Laboratory Animals” of China (D.N. 55, 2001).

### Preparation and characterization of CUR&BBR/GA-HA-Lip

In this study, DSPE-PEG_2000_-HA conjugate was synthesized by one-step coupling reaction between DSPE-PEG_2000_-NH_2_ and HA (MW 5000). DSPE-PEG_2000_-GA was obtained by a two-step process according to our previous research (Jiang et al., 2019). CUR&BBR/GA-HA-Lip was prepared by an ethanol injection method. Briefly, a mixture of PC (120 mg), cholesterol (40 mg), DSPE-PEG-GA (3 mg), and DSPE-PEG-HA (3 mg) were dissolved in 2 ml of absolute ethanol, followed by the addition of CUR and BBR at a ratio of 1:1. Then, the mixed solution was injected into citric acid buffer. The ethanol was evaporated, and citric acid buffer was added to adjust the volume of final liposome suspension to 5 ml. After 5 min of ultrasound, the liposomes were obtained by 15 times extrusion through 200-nm membranes using an extruder for further study. CUR&BBR/Lip and CUR&BBR/GA-Lip were prepared as the control. The un-encapsulated drugs were removed by the Sephadex G50 column, and the absorbance of CUR and BBR was detected by ultraviolet spectrophotometer at 425 and 345 nm, respectively. The loading efficiency (LE) and encapsulation efficiency (EE) were calculated as follows:

LE = (the drug loaded in Lip/total weight of Lip) × 100%
EE = (the drug loaded in Lip/total drug added to system) × 100%

The drug-loaded liposomes were suspended in deionized water, and the characteristics, such as the particle size, polydispersity (PDI), and zeta potentials, were measured using a dynamic light scattering analyzer (Nano-ZS90, Malvern Zetasizer, United Kingdom). The morphology of two liposomal formulations was imaged using a transmission electron microscope (TEM, Hitachi HT7700, Japan).

### The *in vitro* stability and drug release

The colloidal stability was important for the liposome-based drug delivery system. CUR&BBR/GA-HA-Lip was dissolved in PBS (pH = 7.4) or RPMI 1640 medium and kept in the refrigerator at 4°C; the particle size and PDI were monitored once a day for 1 week.

To evaluate the drug release behaviors, we used the dialysis bag diffusion technique to investigate CUR and BBR drug release from CUR&BBR/GA-HA-Lip. Briefly, 1 ml of liposomal solution was placed in the dialysis bag and immersed into 20 ml of the PBS buffer (pH 7.4) containing 1.5% Tween 80 and 20% anhydrous ethanol (Li et al., 2020). The system was kept at 37°C with continuous shaking at 100 rpm. At a predetermined time interval, 1 ml of samples was removed, and the same volume of buffer solution was added. The amounts of released CUR and BBR were calculated using a UV spectrophotometer at 425 and 345 nm, respectively.

### The *in vitro* cellular uptake and drug retention assay

To evaluate the co-targeting properties of GA-HA-Lip, a cellular uptake assay was performed in BEL-7402 and LX-2, respectively. In the study, coumarin-6 (C6), a hydrophobic fluorescent dye, was chosen as a model drug to detect the distribution of the drug in cells, and C6-loaded liposomes were prepared according to the previous method. First, BEL-7402 and LX-2 cells were seeded into a glass-bottom cell-culture dish (ϕ20 mm) for 24 h at 37°C. Then, C6/Lip, C6/GA-Lip, and C6/GA-HA-Lip (equivalent C6 concentration of 1 μg/ml) were added to different wells, respectively. After 2 h of incubation, all reagents were removed, and the cells were fixed with 4% polyformaldehyde for 10 min. Finally, the nuclei were stained with DAPI (0.1 μg/ml) for 8 min; then, the cells were observed using a confocal laser scanning microscope (CLSM).

Recent research work revealed that CAFs could reduce drug retention by upregulating the expression level of MDR-related proteins in TME. To evaluate drug retention of tumor cells, “BEL-7402+LX-2” mixed cells (v:v = 5:1) were seeded in a 24-well plate and cultured for 24 h. Free C6, C6+BBR, C6&BBR/Lip, and C6&BBR/GA-HA-Lip (equivalent BBR concentration of 1 μg/ml) were added for further incubation. After fixation with 4% paraformaldehyde, the mixed cells were stained with DAPI and observed under a CLSM.

### The *in vitro* cytotoxicity assay

The cytotoxicity of different drug formulations against BEL-7402 cells and “BEL-7402+LX-2” mixed cells (v:v = 5:1) was evaluated using the MTT assay. First, BEL-7402 cells and mixed cells were seeded into a 96-well plate at a density of 8,000 cells per well and grown for 24 h to reach the confluence of 80%. Then, the cell culture media were replaced with different concentrations of drug formulations (0.1, 1, 2, 5, and 10 μg/ml of CUR) and kept in incubation at 37°C in 5% CO_2_ environment. After 48 h, 10 μl of MTT (5 mg/ml) solution was added to each well for another 4 h, followed by the addition of dimethyl sulfoxide (DMSO) to dissolve the insoluble formazan crystal. Finally, the absorbance of each well was recorded at 470 nm using a microplate reader.

Furthermore, a live/dead cell viability assay was performed in the mixed cell model. To distinguish LX-2 cells from tumor cells, LX-2 cells were stained with carboxyfluorescein diacetate succinimidyl ester (CFSE). Briefly, 5 μM/ml of CFSE was incubated with LX-2 cells for 30 min to obtain CFSE-labeled LX-2 cells (C-LX-2). The mixed cell suspension of BEL-7402 and C-LX-2 cells at the ratio of 5:1 was seeded in 96-well plates. After 48 h of drug treatment, the culture medium was removed, and PI was added to each well for 15 min, followed by DAPI staining for 8 min. Finally, the cells in each well were observed using a fluorescence microscope, in which, the living LX-2 cells, dead LX-2 cells, and dead BEL-7402 cells were green-, yellow-, and red-colored, respectively.

### *In vivo* biodistribution analysis

To further evaluate the biodistribution of GA&HA-modified liposomes, an advanced HCC mouse model was established. In brief, H22 cells (2 × 10^7^ cells in 100 μl PBS) were implanted on the right flank of female BALB/c mice to establish H22-cell tumor-bearing mice. When the tumor size reached 100 mm^3^, the mice were intravenously administered with H22 cells (1 × 10^7^ cells in 100 μl PBS) to form lung metastases. After 10 days, the mice were randomly divided into three groups, followed by injection of free DiR, DiR/Lip, and DiR/GA-HA-Lip (40 μg/ml, 0.2 ml) *via* the tail vein. At 1, 2, 4, 8, 12, 24, and 48 h, a real-time near–infra-red fluorophore (NIRF) technique was applied to detect drug distribution *in vivo*. At 48 h post-administration, the mice were euthanized, and the main organs (heart, liver, spleen, lung, and kidney) and the tumors were harvested for *ex vivo* imaging. The region-of-interest (ROI) was circled around the harvested tissues, and the fluorescence intensity of DiR was measured using the Maestro 3 software.

### *In vivo* anti-tumor activity

To investigate the anti-tumor efficacy of different drug formulations, we established subcutaneous “H22+m-HSC” tumor-bearing mice models, which were inoculated with H22 and m-HSC cells at a ratio of 5:1 (Xu et al., 2018). When the tumor volume grew to approximately 200 mm^3^, the mice were randomly assigned to six groups (*n* = 5). The mice were injected with free CUR, CUR+BBR, CUR&BBR/Lip, CUR&BBR/GA-Lip, and CUR&BBR/GA-HA-Lip every 2 days at an equivalent dose of CUR (5 mg/kg). The tumor sizes and body weights were measured every 2 day. The tumor volume was calculated based on the following equation: tumor volume = length × (width)^2^/2 (Li et al., 2020). After 14 days, the mice were euthanized, and the tumor weight was measured. The tumor inhibition rate (IR) was calculated using the following equation: IR = (1-the tumor weight of treatment group/the tumor weight of saline group) × 100% (Wang et al., 2020). The tumors were fixed with 4% neutral paraformaldehyde for pathological analysis. Moreover, the Masson’s staining, immunofluorescence, and immunohistochemistry assay were performed to investigate the change in the TME.

### Statistical analysis

The experiments were performed at least three times, and the data are shown as mean ± standard deviation (SD). A Student’s *t*-test or one-way ANOVA was used for statistical analysis, and *p* < 0.05 was considered to show statistical significance.

## Results and discussion

### Characteristics of CUR&BBR/GA-HA-Lip

GA-HA-Lip liposomes were prepared for co-delivery of CUR and BBR by ethanol injection, in which GA and HA were chosen as targeting ligands. The physicochemical properties of different liposome formulations are shown in Table 1. The particle size of CUR&BBR/GA-HA-Lip was 159.39 ± 3.16 nm with a narrow size distribution (PDI < 0.2), and the zeta potential was negative. The favorable characterizations of nano-sized carriers play an important role in prolonging the circulation time of liposomes in blood and improving drug accumulation by the enhanced permeability and retention effect (EPR). The encapsulation efficiency (EE) of both CUR and BBR exceeded 90%, indicating that the two drugs could be effectively entrapped in liposomes. The loading content (LC) was similar for CUR (2.15%) and BBR (2.13%). The morphology of blank liposome and CUR&BBR/GA-HA-Lip were uniformly spherical (Figures 2A,B).

**TABLE 1:** Characteristics of different liposomal formulations.

Formulation	DLS (nm)	PDI	ζ-Potential (mV)	EE%	LC%
Blank lip	146.64 ± 0.72	0.15 ± 0.01	−2.01 ± 0.07	—	—
CUR&BBR/Lip	150.17 ± 2.36	0.16 ± 0.03	−1.02 ± 0.05	96.69 ± 2.33 (CUR) 94.91 ± 0.48 (BBR)	2.32 ± 0.08 (CUR 2.24 ± 0.04 (BBR)
CUR&BBR/GA-Lip	151.06 ± 8.21	0.17 ± 0.02	−0.35 ± 0.21	96.82 ± 1.75 (CUR) 95.91 ± 1.04 (BBR)	2.27 ± 0.04 (CUR) 2.24 ± 0.03 (BBR)
CUR&BBR/GA-HA-Lip	159.39 ± 3.16	0.17 ± 0.01	−0.24 ± 0.35	93.66 ± 3.08 (CUR) 92.59 ± 5.45 (BBR)	2.15 ± 0.07 (CUR) 2.13 ± 0.13 (BBR)

All results are the mean values of the measurements from three replicates. The values are expressed as mean ± SD.

**FIGURE 1:**
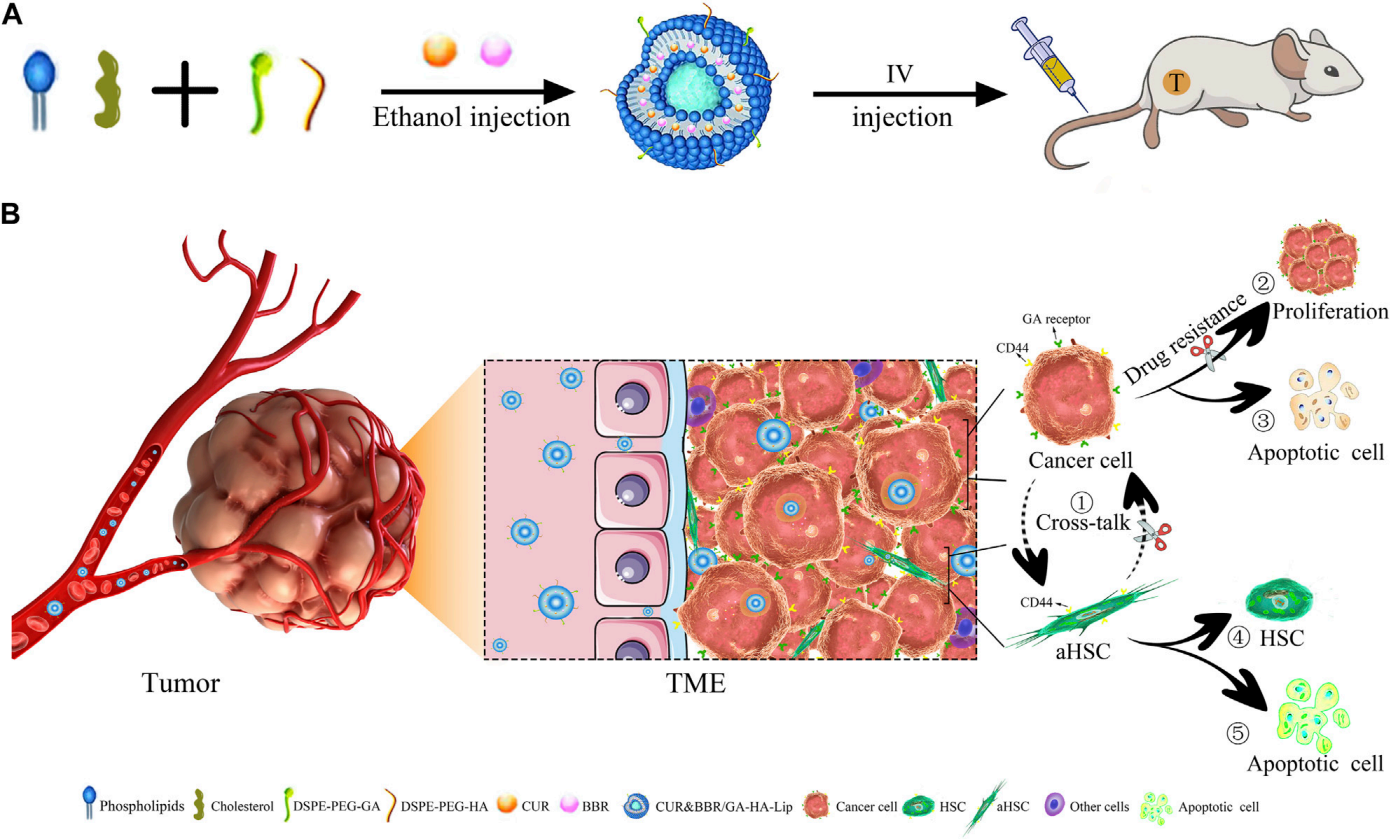
| Anti-tumor schematic diagram of CUR&BBR/GA-HA-Lip. **(A)** The preparation and drug administration of the liposomes. **(B)** The anti-tumor activities of the combined therapy of CUR and BBR. ① Blocking the cross-talk between aHSCs and tumor cells, ②–③ inhibiting proliferation and inducing apoptosis of tumor cells, and ④–⑤ inhibiting the activation of HSCs.

**FIGURE 2:**
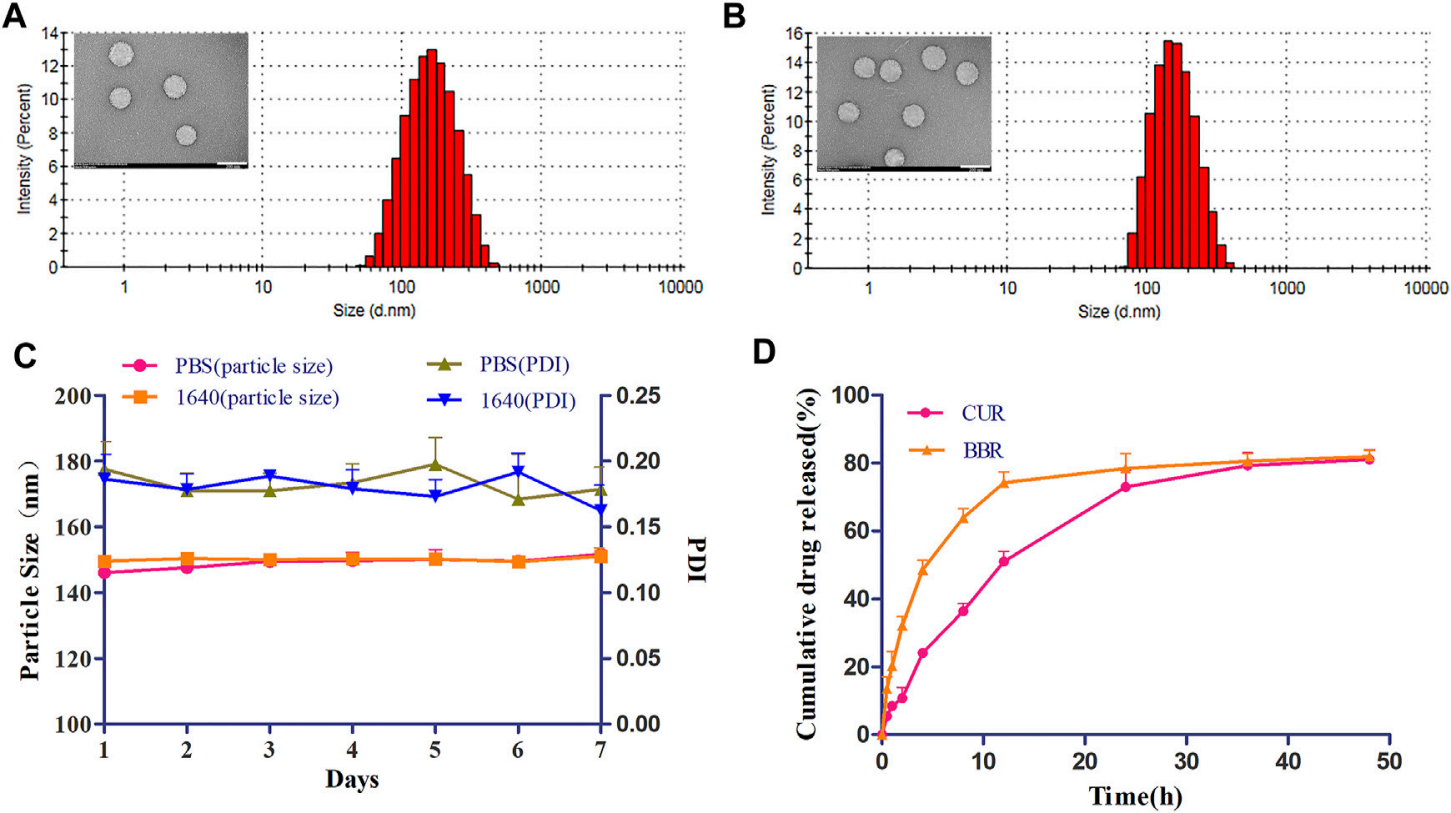
Physicochemical characterization of CUR&BBR/GA-HA-Lip. Particle size and TEM images of blank liposome **(A)** and CUR&BBR/GA-HA-Lip **(B)**. Scale bar, 200 nm. **(C)** Stability of CUR&BBR/GA-HA-Lip within 7 days by monitoring the particle size and PDI. **(D)** Drug release profiles of CUR&BBR/GA-HA-Lip.

The stability of CUR&BBR/GA-HA-Lip was evaluated in RPMI 1640 medium and PBS at 4°C. As shown in Figure 2C, no significant change in particle size and PDI was observed in 1 week, indicating that CUR&BBR/GA-HA-Lip exhibited high stability, and that GA-HA-Lip was a potential nano-carrier for delivery of CUR and BBR. The release profiles of CUR and BBR were investigated by the dialysis method. Figure 2D shows that both CUR and BBR exhibited time-dependent drug release from liposomes within 48 h. There was no obvious burst release of the two drugs in the first 4 h. At 48 h, the cumulative drug release of CUR and BBR was 81.09% and 81.93%, respectively, suggesting that the majority of both CUR and BBR could effectively escape from liposomes into the release medium.

### *In vitro* cellular uptake and drug detention studies

**FIGURE 3:**
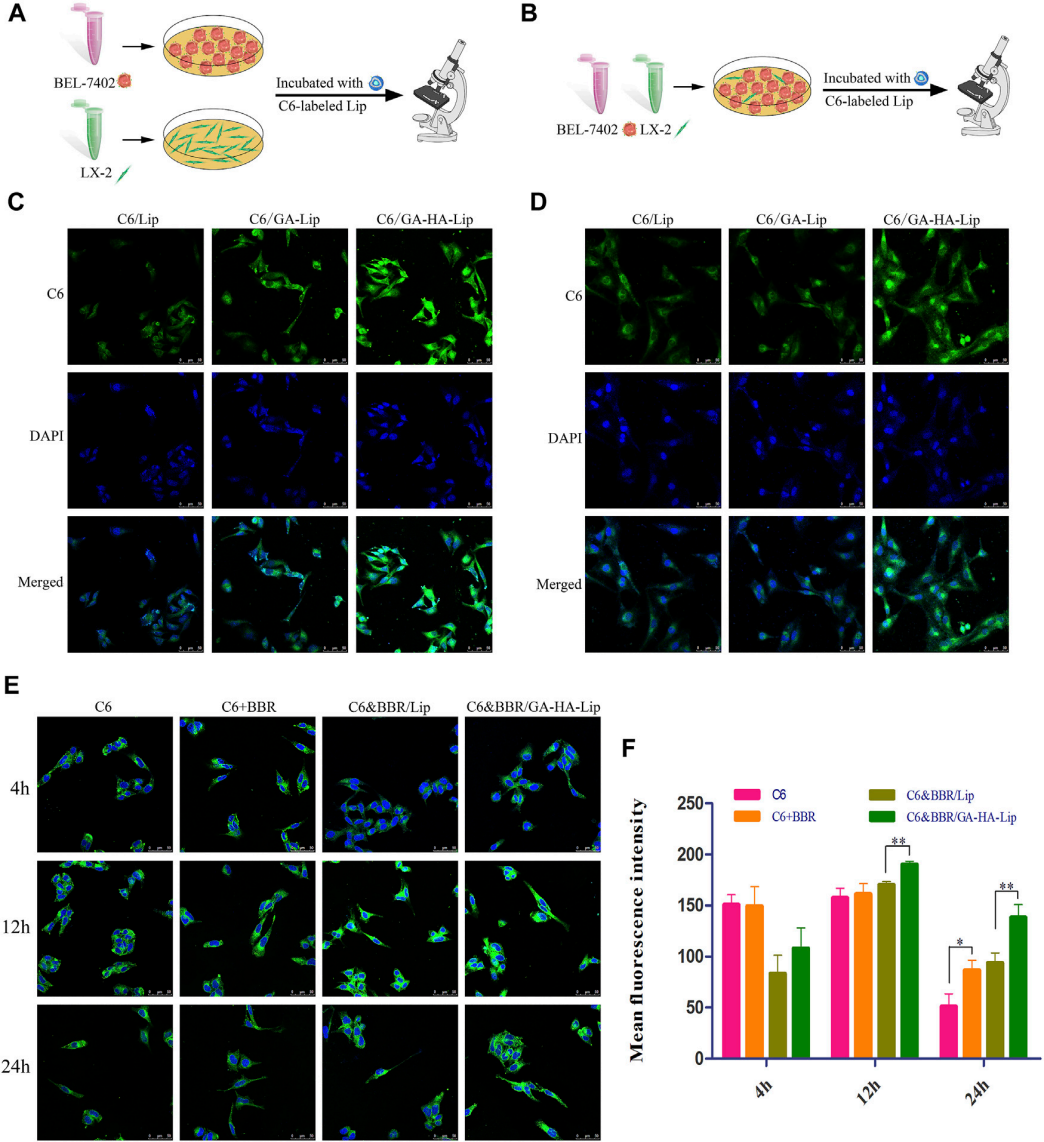
*In vitro* cellular uptake and drug detention studies in a co-cultured system. **(A)** Cell uptake model of BEL-7402 and LX-2 cells. **(B)** Drug retention assay model. CLSM images of BEL-7402 cells **(C)** and LX-2 cells **(D)** treated with C6/Lip, C6/GA-Lip, and C6/GA-HA-Lip at 2 h, where the nuclei were labeled with DAPI (blue) and Lip were labeled with C6 (green); scale bars represent 50 μm. **(E)** Confocal microscopy and **(F)** quantitative analysis of drug retention were used to detect drug retention at 4, 12, and 24 h; scale bar represents 50 µm.

GA-modified liposomes could deliver drugs to HCC cells through GA-receptor–mediated endocytosis, but had no obvious effect on aHSCs (CAF phenotype) in our previous research. Recent studies have shown that the CD44 receptor is upregulated in the aHSCs. Hence, we prepared GA&HA-modified liposomes to target aHSCs and HCC cells simultaneously. Interestingly, as shown in Figure 3C, C6/GA-HA-Lip showed stronger drug uptake in BEL-7402 cells than C6/GA-Lip. Furthermore, LX-2 cells were pre-incubated with the condition medium of BEL-7402 cells to obtain aHSCs. In Figure 3D, there was no significant difference in fluorescence intensity between the C6/Lip and C6/GA-Lip. However, fluorescence intensity in C6/GA-HA-Lip was significantly greater than that in C6/GA-Lip, suggesting that the introduction of HA molecules promoted drug uptake of aHSCs. Therefore, the GA/HA-modified liposomes could target tumor cells and aHSCs simultaneously.

Considering that TME was composed of tumor cells and other stromal cells, traditional tumor cell model is insufficient to simulate the real TME. A drug detention assay was performed in “BEL-7402+LX-2”co-culture to determine the amount of drugs in the tumor cells (Figure 3B). As shown in Figures 3E,F, compared with free C6, stronger fluorescence signals were observed in the three combined drug groups, indicating that the addition of BBR reduces drug efflux. The possible reason was that BBR could reduce the cross-talk between LX-2 and BEL-7402 cells by inhibiting the activation of HSCs. Interestingly, there were greater fluorescence in C6&BBR/GA-HA-Lip than C6&BBR/Lip. The result might be due to the fact that the modification of HA and GA improved cellular uptake of LX-2 and BEL-7402 through CD44- and GA-receptor–mediated endocytosis, respectively, leading to higher drug retention at tumor cells.

### *In vitro* cytotoxicity assays

**FIGURE 4:**
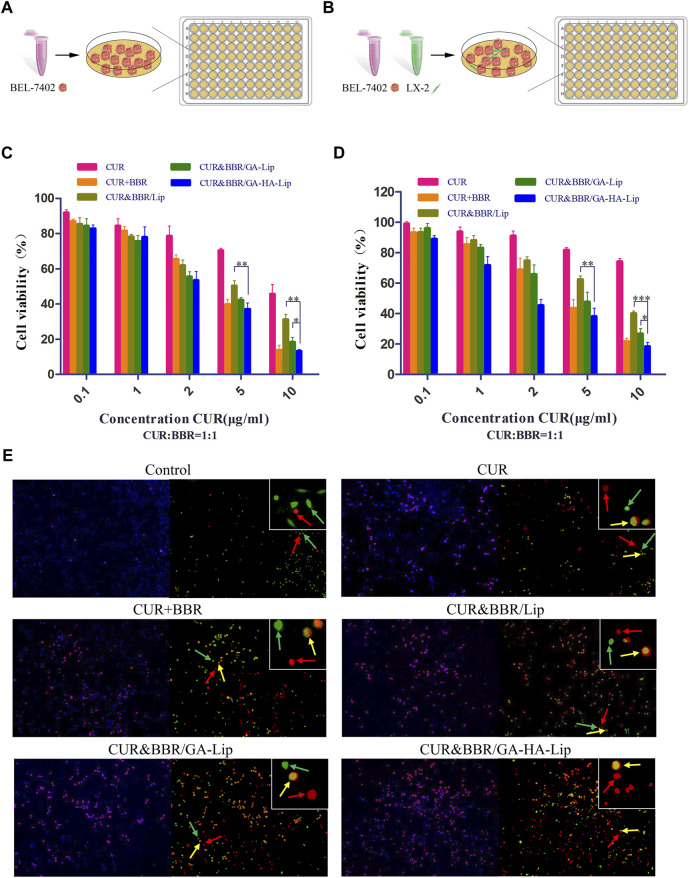
*In vitro* cytotoxicity analysis. *In vitro* research model of BEL-7402 cells **(A)** and BEL-7402+LX-2 co-cultured system **(B)**. Cytotoxicity of different drug formulations against BEL-7402 cells **(C)** and BEL-7402+LX-2 co-cultured system **(D)**. **(E)** Live/dead staining assay against co-cultured system. Green fluorescence: live LX-2 cells; red fluorescence: dead BEL-7402 cells; yellow fluorescence: dead LX-2 cells.

The cytotoxicity of different treatment groups against BEL-7402 cells was evaluated using the MTT assay (Figure 4A). As shown in Figure 4C, the cell viability in the three combined drug groups was lower than that in the single drug groups, indicating that combination therapy of CUR and BBR exhibited higher cytotoxicity. Compared with CUR&BBR/Lip, CUR&BBR/GA-HA-Lip showed greater anti-proliferative effect. The result might be due to the fact that the modification of GA and HA improved cellular uptake of Lip, leading to strong cytotoxicity.

In the actual TME, aHSCs play an important role in tumor development by regulating the cross-talk between aHSCs and tumor cells (Gao et al., 2021). The traditional tumor cell model could not effectively evaluate the anti-proliferation effect of drugs in TME. We established a co-cultured cell model, in which BEL-7402 and LX-2 cells were mixed at a ratio of 5:1 (Figure 4B). In Figure 4D, compared with BEL-7402 cells alone, the half-inhibitory concentration (IC50) of CUR against the co-cultured model was 40.77 μg/ml, which was 2.91-fold higher than that in BEL-7402 cells, suggesting that LX-2 cells decreased the sensitivity of BEL-7402 cells to CUR. As expected, the combined formulation based on GA-HA-Lip showed stronger anti-proliferation effect than other groups. The possible reason was that CUR&BBR/GA-HA-Lip could be taken up by BEL-7402 cells and LX-2 by GA-receptor– and CD44-receptor–mediated endocytosis, respectively, leading to greater anti-tumor effect. The cytotoxicity of different drug formulations were also evaluated by a live/dead cell viability assay, in which green, yellow, and red spots represented live HSCs, dead HSCs, and dead tumor cells, respectively. As shown in Figure 4E, more red and yellow dots were observed in the cells treated with CUR&BBR/GA-HA-Lip than other groups, indicating that GA-HA-Lip promoted pro-apoptotic effect of drugs.

### The *in vivo* biodistribution analysis

**FIGURE 5:**
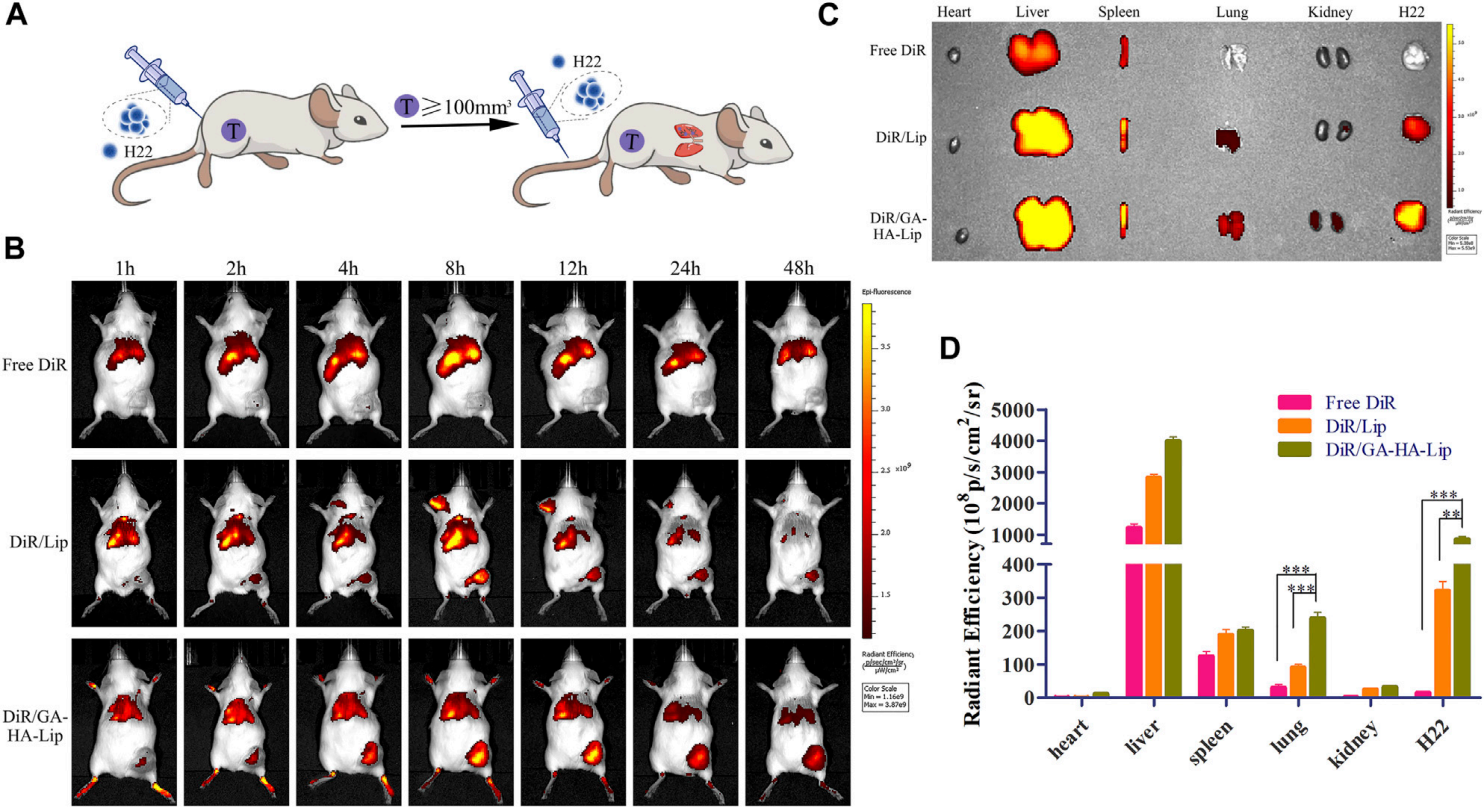
The *in vivo* biodistribution of GA-HA-Lip. **(A)** Establishment of a novel advanced HCC mice model. **(B)** Fluorescence imaging at different time points. **(C)** *Ex vivo* IVIS images at 48 h. **(D)** The fluorescent intensity of different tissues.

It was difficult to improve the long-term survival rate of patients with advanced HCC due to distant metastasis of tumor cells. In this study, an advanced HCC mice model was established to investigate the drug biodistribution *in vivo* (Figure 5A), and NIRF images were obtained at different time points after injection of different formulations. As shown in Figure 5B, compared with the free DiR group, the fluorescent signals in the tumor region of the liposomal groups were stronger, suggesting that liposomes could promote drug accumulation in tumors. Notably, DiR/GA-HA-Lip exhibited greater fluorescent intensity in tumors than DiR/Lip. The possible explanation was that the liposomes modified by GA and HA molecules improved cellular internalization *via* GA- and/or HA-receptor–mediated manner, leading to higher DiR accumulation in tumor. At 48 h, the tumor and major organs were harvested for further *ex vivo* imaging analysis. In Figures 5C,D, intratumoral fluorescence intensities in DiR/Lip and DiR/GA-HA-Lip groups were 21.01- and 55.42-fold higher than that in free DiR, respectively. Interestingly, stronger fluorescent signals were detected in the lung of DiR/GA-HA-Lip group than in that of other groups. Given that HCC cells could metastasize to lung tissues, the result suggested that GA-HA-Lip could accumulate not only in primary tumor but also in the metastasis area of lung. In addition, due to loss of aHSCs in this H22-bearing mice model, the targeting effect of GA-HA-Lip depends mainly on nano-sized carrier-derived EPR effect and GA/GA receptor-mediated endocytosis.

### The *in vivo* anti-tumor activity

**FIGURE 6:**
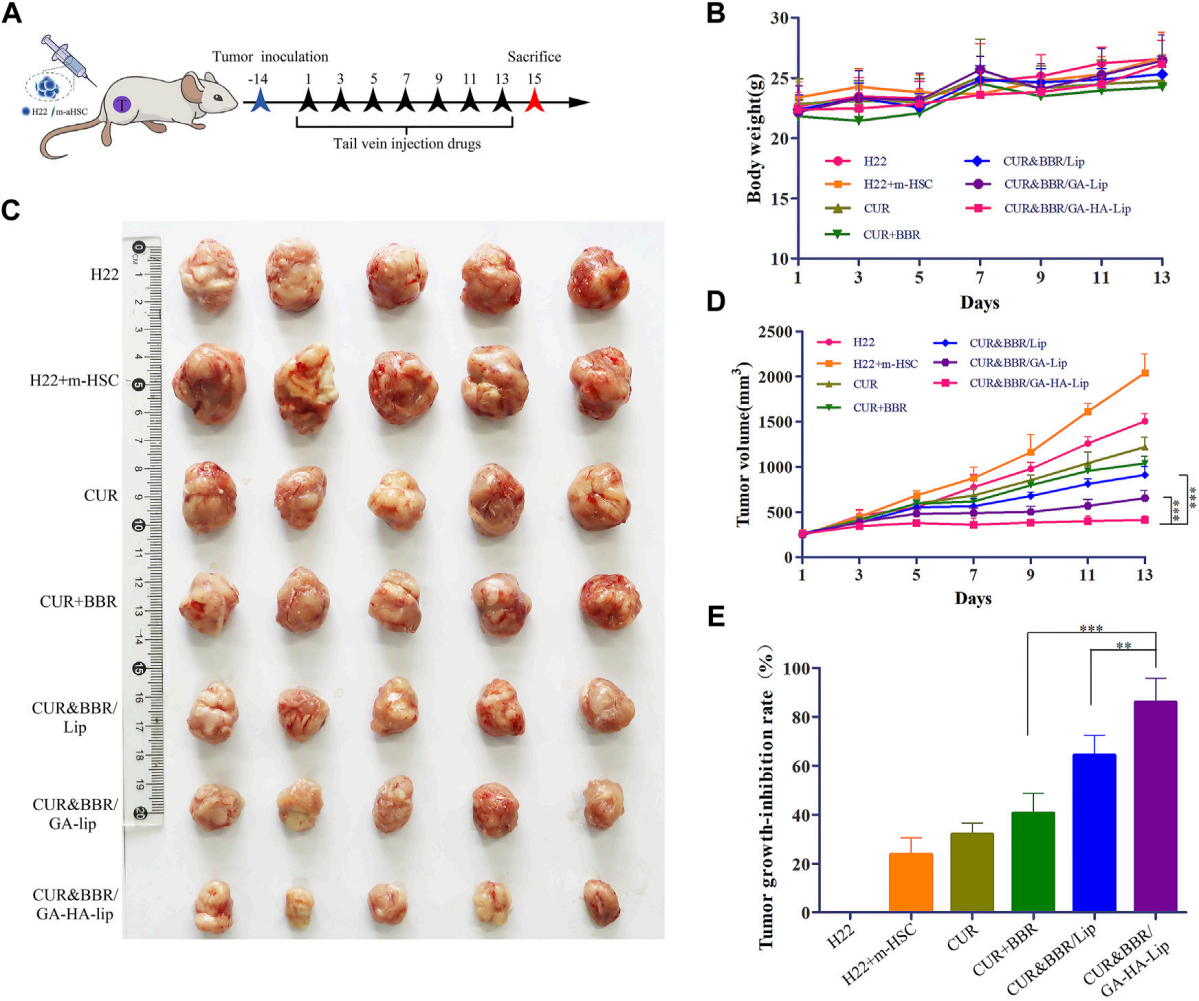
The anti-tumor evaluation of the H22+m-HSC tumor-bearing mice model. **(A)** Scheme of the strategy for the H22+m-HSC tumor-bearing mice model. **(B)** Body weight. **(C)** Images of tumor tissue in different treatment groups. **(D)** The curve of tumor volume during treatment. **(E)** Analysis of the tumor inhibition rate of different preparations.

**FIGURE 7:**
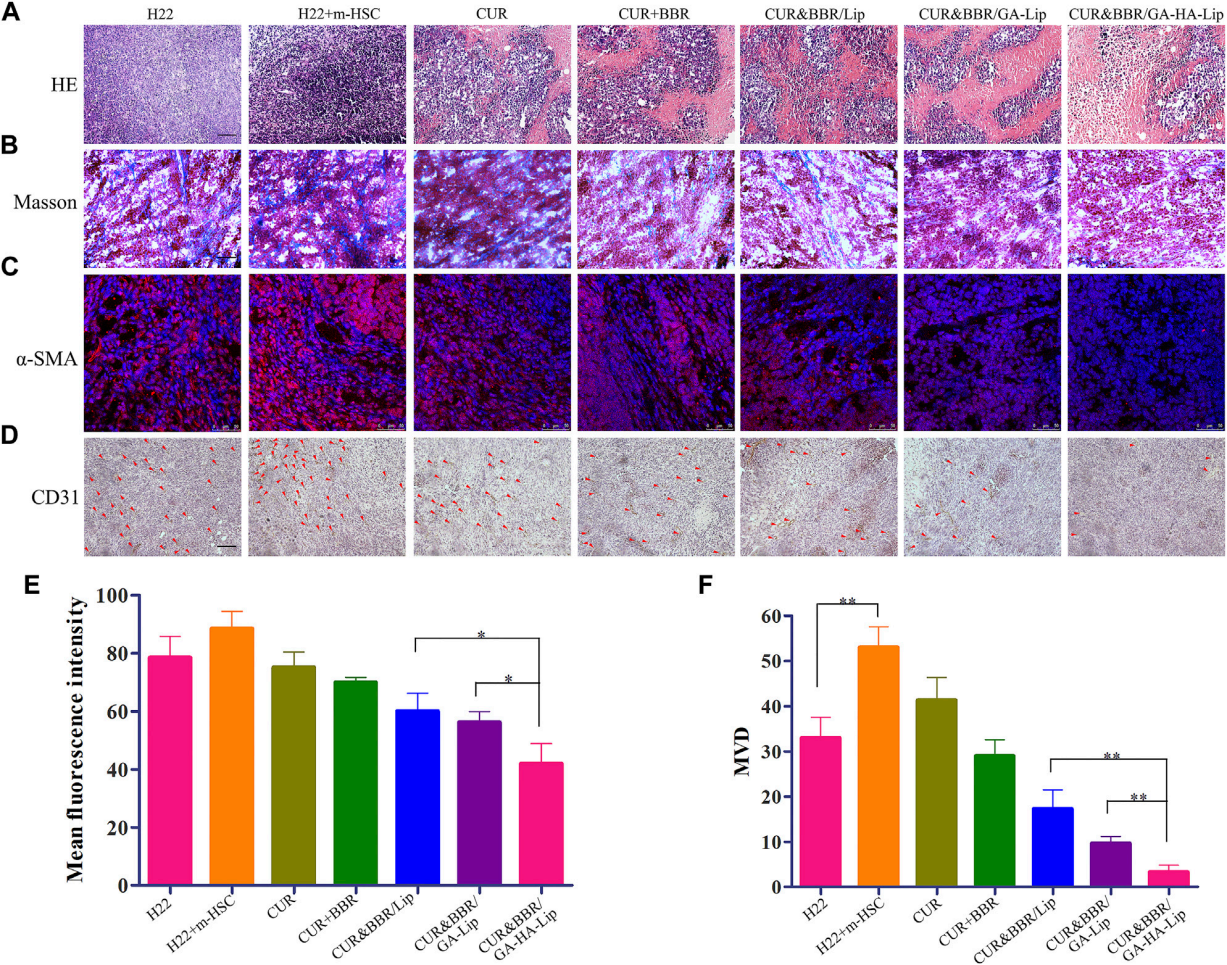
The histological analysis of the H22+m-HSC tumor-bearing mice model. **(A)** Histological analysis of tumors with H&E staining; scale bars represent 200 µm. **(B)** Masson stain of tumor tissue; scale bars represent 200 µm. **(C)** IF staining of α-SMA; scale bars represent 50 µm. **(D)** IHC staining of CD31; scale bars represent 200 µm. **(E)** Quantitative analysis of α-SMA and **(F)** CD31.

Considering that aHSCs could promote tumor development by the cross-talk between aHSCs and tumor cells in the TME, the traditional subcutaneous H22 cell–bearing mouse model was difficult to really evaluate the anti-tumor effect of drugs. To mimic the TME *in vivo*, we established a novel co-bearing mouse model by implanting both m-HSC and H22 cells (the ratio of H22 to m-HSC was 5:1) (Figure 6A). The body weight and tumor volume of the mice were measured every other day. As shown in Figure 7B, no significant difference was observed between liposomal formulations and the saline group. Interestingly, in Figures 6C,D, compared with H22 cell–bearing mice, the tumors in “H22+m-HSC” cell–bearing mice were significantly greater, indicating that m-HSCs might promote the tumor growth. Given that there were amounts of aHSCs in actual TME, the “H22+m-HSC” cell–bearing mice model was a potential model for anti-tumor evaluation *in vivo*. After drug injection, the tumor growth could be inhibited in all treatment groups. While the three liposomal formulations exhibited lower tumor size than free mixture of CUR and BBR in Figure 6C, suggesting that liposomes could enhance anti-tumor effect of the drugs. As shown in Figure 6E, compared with CUR&BBR/Lip, the combination therapy based on GA-HA-Lip showed higher tumor growth inhibition rate (85.93%) and larger necrotic region. This might be due to the fact that GA-HA-Lip could be taken up by aHSCs and tumor cells simultaneously, leading to greater anti-proliferation effect.

### The histological analysis

Recent research work showed that aHSCs could promote collagen fiber deposition and angiogenesis in the TME (Affo et al., 2017). To examine the change in the TME, immunofluorescence, Masson’s staining, and immunohistochemistry assay were performed (Zhao et al., 2020). In Figure 7A, notable karyolysis and cytoplasmic vacuolations were observed in all drug-treated groups. As expected, CUR&BBR/GA-HA-Lip exhibited greater pro-apoptotic effect than other groups. As shown in Figure 7B, compared with other groups, the collagen fibers (blue spots) in the CUR&BBR/GA-HA-Lip group was significantly reduced, indicating stronger inhibition of ECM deposition. Furthermore, in Figures 7C,E, more red spots were observed in the “H22+m-HSC” mouse model than in the H22 mouse model, indicating that HSCs were activated in the mouse model. After drug treatment, the fluorescent signals decreased significantly in all the drug treatment group, and almost disappeared in the CUR&BBR/GA-HA-Lip group. The result suggested that CUR&BBR/GA-HA-Lip could effectively inhibit the activation of HSCs. Tumor angiogenesis has been proven to promote tumor development (Dasgupta et al., 2015). To investigate the anti-tumor efficacy of combination therapy, the MVD assay was performed through immunohistochemistry analysis of CD31 protein. In Figures 7D,F, fewer CD31-positive microvessels were observed in CUR&BBR/GA-HA-Lip than in other groups, indicating that GA-HA-Lip promotes anti-angiogenic activity.

## Conclusion

Recent efforts have been focused on combined pro-apoptotic and anti-CAF activities to improve anti-tumor effect of drug. In this study, GA&HA-modified liposomes co-loaded with curcumin and berberine (CUR&BBR/GA-HA-Lip) were prepared to inhibit drug resistance and proliferation of HCC cells. The GA-HA-Lip could be taken up by tumor cells and aHSCs simultaneously and reduce drug resistance and inhibit tumor cell proliferation. To mimic the actual TME, “BEL-7402+HSCs” co-cultured cell model and “H22+m-HSCs” co-implanted mouse model were established to evaluate the anti-tumor effect of different drug formulations. The results showed that the addition of HSCs in these models could induce drug resistance of tumor cells. The anti-tumor studies showed that CUR&BBR/GA-HA-Lip could promote the anti-proliferation effect of tumor cells, inhibit the activation of HSCs, and reduce ECM deposition and tumor angiogenesis. In summary, CUR&BBR/GA-HA-Lip achieves synergistic effects by combining pro-apoptotic and anti-aHSCs activities, and provides a promising strategy for HCC treatment.

## Data Availability

The raw data supporting the conclusion of this article will be made available by the authors, without undue reservation.
